# Pharmacological targeting the ATR–CHK1–WEE1 axis involves balancing cell growth stimulation and apoptosis

**DOI:** 10.18632/oncotarget.2508

**Published:** 2014-09-25

**Authors:** Joyce P.Y. Mak, Wing Yu Man, Hoi Tang Ma, Randy Y.C. Poon

**Affiliations:** ^1^ Division of Life Science, Center for Cancer Research, and State Key Laboratory of Molecular Neuroscience, Hong Kong University of Science and Technology, Clear Water Bay, Hong Kong

**Keywords:** anticancer drugs, checkpoint, DNA damage, mitosis, mitotic catastrophe

## Abstract

The ATR–CHK1–WEE1 kinase cascade's functions in the DNA damage checkpoints are well established. Moreover, its roles in the unperturbed cell cycle are also increasingly being recognized. In this connection, a number of small-molecule inhibitors of ATR, CHK1, and WEE1 are being evaluated in clinical trials. Understanding precisely how cells respond to different concentrations of inhibitors is therefore of paramount importance and has broad clinical implications. Here we present evidence that in the absence of DNA damage, pharmacological inactivation of ATR was less effective in inducing mitotic catastrophe than inhibition of WEE1 and CHK1. Small-molecule inhibitors of CHK1 (AZD7762) or WEE1 (MK-1775) induced mitotic catastrophe, as characterized by dephosphorylation of CDK1^Tyr15^, phosphorylation of histone H3^Ser10^, and apoptosis. Unexpectedly, partial inhibition of WEE1 and CHK1 had the opposite effect of accelerating the cell cycle without inducing apoptosis, thereby increasing the overall cell proliferation. This was also corroborated by the finding that cell proliferation was enhanced by kinase-inactive versions of WEE1. We demonstrated that these potential limitations of the inhibitors could be overcome by targeting more than one components of the ATR–CHK1–WEE1 simultaneously. These observations reveal insights into the complex responses to pharmacological inactivation of the ATR–CHK1–WEE1 axis.

## INTRODUCTION

Cyclin-dependent kinase 1 (CDK1) is the major kinase for driving cells into mitosis. A defining characteristic of CDK1 activation is a system of feedback loops that converts the progressive accumulation of its activating partner cyclin B1 into an abrupt activation of CDK1 [1]. Premature activation of cyclin B1–CDK1 complexes is prevented by a mechanism involving inhibitory phosphorylation of CDK1^Thr14/Tyr15^ by MYT1 and WEE1. At the end of G_2_, the stockpile of inactive cyclin B1–CDK1 complexes is rapidly activated by CDC25 phosphatases. Cyclin B1–CDK1 catalyzes its own activation with feedback loops that activate CDC25 and inactivate WEE1/MYT1.

WEE1 is a dual-specificity kinase that phosphorylates CDK1^Tyr15^ (but not CDK1^Thr14^) [2]. MYT1, a kinase that normally bound to the endoplasmic reticulum and Golgi complex, can phosphorylate both the Thr14 and Tyr15 [3,4]. While WEE1 plays an indispensable role in controlling the timing of mitosis, MYT1 appears to play a relatively minor role in the somatic cell cycle [5].

A surveillance mechanism termed the G_2_ DNA damage checkpoint prevents entry into mitosis after DNA damage. The checkpoint comprises of a kinase cascade initiating with the activation of ATM and the related ATR. Activated ATM/ATR then phosphorylates residues in the SQ/TQ domain of CHK1 and CHK2, stimulating the activity of these effector kinases [6]. CHK1/CHK2 subsequently acts on all three isoforms of the CDC25 family to suppress their activities [1]. CHK1 also phosphorylates and activates WEE1 by promoting 14-3-3 binding [7,8]. Suppression of CDC25 or activation of WEE1 tips the balance towards CDK1^Thr14/Tyr15^ phosphorylation, thereby preventing damaged cells from entering mitosis. Although there are considerable overlaps in the ATM/ATR–CHK1/CHK2 axis, it is generally believed that while the ATM–CHK2 pathway primarily responds to DNA double-strand breaks, the ATR–CHK1 pathway is activated by a broader spectrum of DNA abnormalities [9]. Premature inactivation of the checkpoint promotes a process often termed mitotic catastrophe, which is characterized by precocious mitosis followed by apoptosis or mitotic slippage [10].

Agents that cause replication stress also activate a similar checkpoint involving ATR–CHK1–WEE1. ATR is activated after recruited to the single-strand binding protein RPA that coats ssDNA, thereby stabilizing the stalled forks and initiating checkpoint activation [11]. Origin firing, replication forks progression, and mitosis are suppressed by this checkpoint.

In addition to its role in checkpoint control, the ATR–CHK1–WEE1 pathway also plays an essential role in the unperturbed cell cycle. Deletion of ATR [12,13], CHK1 [14], or WEE1 [15] resulted in embryonic lethality. Inhibition of these kinases during normal S phase facilitates an unscheduled activation of cyclin E–CDK2. The resulting increase in initiation of DNA replication promotes DNA damage in a yet incompletely understood mechanism [16]. One possibility is that the unscheduled initiation of dormant origins reduces cellular resources such as dNTPs or histone chaperones to levels insufficient to support the number of active replication forks, thereby leading to replication stalling and SLX4/MUS81-mediated DNA double-strand breakage [17] [18].

A promising anticancer strategy is by ablating the G_2_ DNA damage checkpoint through targeting the ATR–CHK1–WEE1 pathway. A number of small-molecule inhibitors of ATR, CHK1, and WEE1 are being evaluated in clinical trials, mainly in combination with DNA-damaging agents. On the other hand, it is possible that these inhibitors can be effective as monotherapeutic agents without DNA damage. Establishing precisely how cells respond to different concentrations of inhibitors is therefore of crucial importance. Based on these premises, we found that in the absence of DNA damage, inhibition of ATR was less useful in inducing mitotic catastrophe comparing to inhibition of WEE1 and CHK1. Unexpectedly, sublethal concentrations of inhibitors of WEE1 and CHK1 in fact accelerated the cell cycle and increased cell proliferation. We demonstrated that combinatorial treatment of inhibitors targeting the ATR–CHK1–WEE1 pathway may be an alternative and effective strategy in inducing mitotic catastrophe without using DNA damage.

## RESULTS

### Pharmacological inactivation of CHK1 and WEE1 but not ATR induces mitotic catastrophe

**Figure 1:**
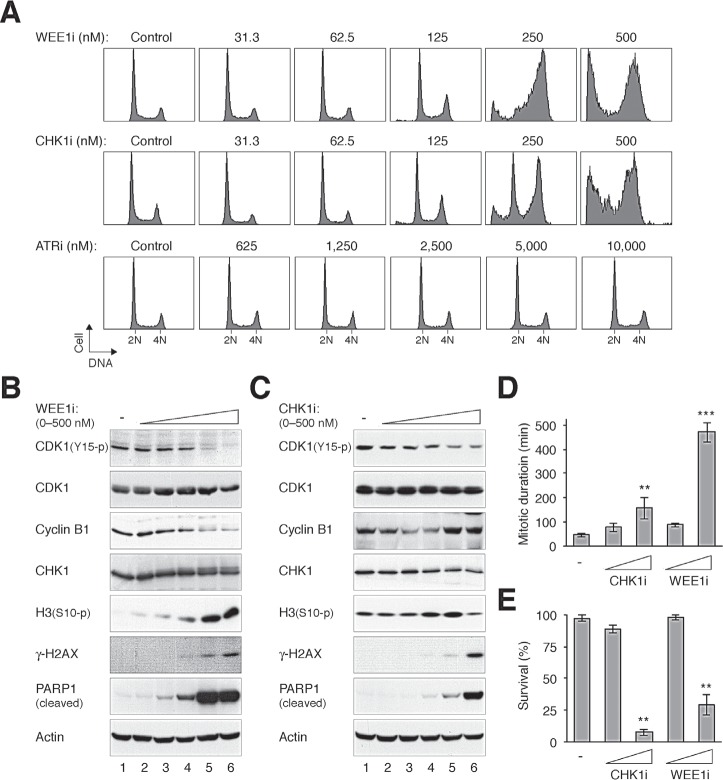
Differential effects of targeting components of the ATR–CHK1–WEE1 cascade (A) Inhibition of CHK1, WEE1, but not ATR disrupts the cell cycle. HeLa cells were incubated with either buffer or the indicated concentrations of MK-1775 (WEE1i), AZD7762 (CHK1i), or VE-821 (ATRi). After 24 h, the cells were harvested and analyzed with flow cytometry. The positions of 2N and 4N DNA content are indicated. Note that a higher range of ATRi concentration was used. (B) WEE1i induces premature mitosis and apoptosis. HeLa cells were incubated with either buffer or different concentrations of WEE1i as described in panel (A). Lysates were prepared and the expression of the indicated proteins was analyzed with immunoblotting. Actin analysis was included to assess protein loading and transfer. (C) CHK1i induces premature mitosis and apoptosis. HeLa cells were treated with either buffer or different concentrations of CHK1i as described in panel (A). Lysates were prepared and the expression of the indicated proteins was analyzed with immunoblotting. (D) Inhibition of CHK1 and WEE1 increases mitotic duration. HeLa cells expressing histone H2B-GFP were incubated with CHK1i or WEE1i (100 nM or 1 μM). Individual cells were then tracked for 24 h using time-lapse microscopy. The mitotic duration was quantified (mean ±90% CI; *n* = 50). Treatment with 1 μM of CHK1i or WEE1i significantly increased mitotic length (*** *P* < 0.001, ** *P* < 0.01; Student's *t-*test). The detailed data for individual cells are shown in Fig S2. (E) Inhibition of CHK1 and WEE1 reduces survival. Cells were treated with CHK1i or WEE1i and imaged with time-lapse microscopy as described in panel (D). The percentage of cell survival was quantified (*n* = 50). Mean ±SD was calculated from three independent experiments. Treatment with 1 μM of CHK1i or WEE1i significantly reduced survival (** *P* < 0.01; Student's *t-*test).

Given that relatively specific small-molecule inhibitors of components of the ATR–CHK1–WEE1 cascade have been developed, we first examined if they could stimulate similar cell cycle responses in otherwise unstressed cells. Fig 1A shows that incubation of HeLa cells with the WEE1 inhibitor MK-1775 [19] (designated WEE1i herein) or the CHK1 inhibitor AZD7762 [20] (designated CHK1i herein) was sufficient to enrich cells in G_2_/M or the later part of S phase. The appearance of cells possessing sub-G_1_ DNA content after incubation with high concentrations of the chemicals indicated extensive apoptosis was induced (Fig 1A).

In marked contrast, an inhibitor of ATR (VE-821 [21], designated ATRi herein) did not induce similar cell cycle delay even when used at 10 μM (250 nM of CHK1i or WEE1i was sufficient to induce G_2_/M defects) (Fig 1A). Similar results were obtained using another cell line (H1299) (Supplemental Fig S1A), excluding the possibility that the differential effects of the chemicals were peculiar for HeLa cells.

Inhibition of either CHK1 or WEE1 resulted in mitotic catastrophe, as indicated by the dephosphorylation of CDK1^Tyr15^ and an accumulation of mitotic markers including phosphorylated histone H3^Ser10^ (Fig 1B and 1C). The cells eventually accumulated DNA damage and underwent apoptosis, as indicated by the appearance of γ-H2AX and cleaved PARP1, respectively. As expected, ATRi did not affect these mitotic and apoptotic events up to 5 μM (Fig S1B).

To attain more direct insights into the fates of CHK1i/WEE1i-treated cells, cells expressing histone H2B-GFP were used and individual cells were tracked with live-cell imaging. Time-lapse microscopy indicated that inhibition of WEE1 (and to a lesser extent CHK1) increased the duration of mitosis (Fig 1D, the data for individual cells are shown in Fig S2). Furthermore, both WEE1i and CHK1i reduced cell survival within the imaging period (Fig 1E).

**Figure 2:**
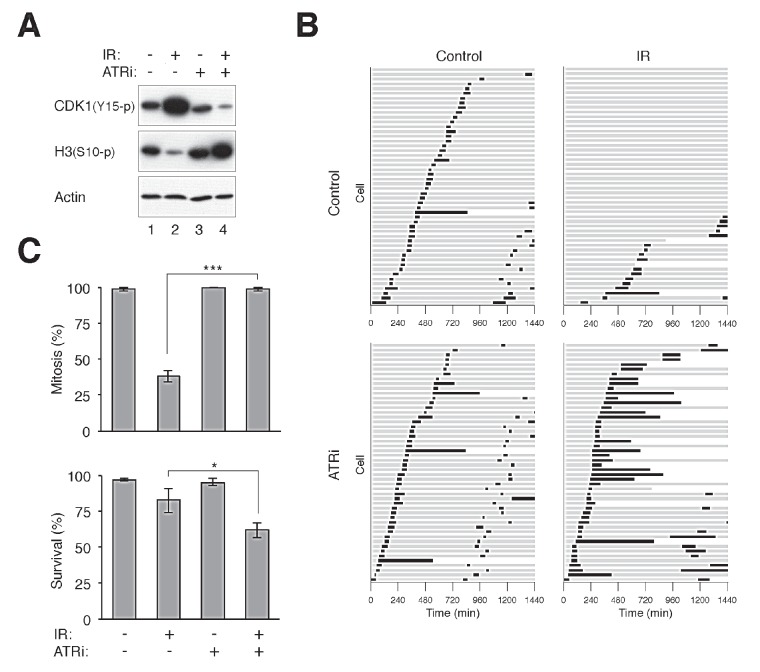
Disruption of the G_2_ DNA damage checkpoint by ATRi (A) Disruption of the DNA damage checkpoint by VE-821. HeLa cells were either untreated or irradiated with 15 Gy of ionizing radiation (IR). After 16 h, the cells were incubated with either buffer or 2.5 μM of VE-821 (ATRi). Nocodazole was also applied to trap cells in mitosis. The cells were harvested after another 6 h. Lysates were prepared and the indicated proteins were detected with immunoblotting. Uniform loading of lysates was confirmed by immunoblotting for actin. (B) Inhibition of ATR bypasses the IR-mediated G_2_ arrest. HeLa cells expressing histone H2B-GFP were either untreated or irradiated with 15 Gy of IR. After 16 h, the cells were incubated with either buffer or ATRi (2.5 μM). Individual cells were then tracked for 24 h with time-lapse microscopy. Each horizontal bar represents one cell (*n* = 50). Grey: interphase; black: mitosis (from DNA condensation to anaphase); truncated bars: cell death. ATRi-treated cells entered the first mitosis significantly faster (*** *P* < 0.001; Student's *t-*test) and more cells were able to undergo the second mitosis during the imaging period. (C) Cells were treated with IR and ATRi and analyzed with live-cell imaging as described in panel (B). The percentages of cells that entered mitosis and survival during the imaging period were quantified (*n* = 50). Mean ±SD was calculated from three independent experiments. Treatment with ATRi significantly promoted mitosis (*** *P* < 0.001) and reduced survival (* *P* < 0.1) in IR-treated cells (Student's *t-*test).

To ensure that the ATRi used was actually capable of inhibiting ATR, cells were first arrested in G_2_ phase with DNA damage before challenged with ATRi (Fig 2A). Activation of the G_2_ DNA damage checkpoint by ionizing radiation was characterized by a high level of CDK1^Tyr15^ phosphorylation and a low level of histone H3^Ser10^ phosphorylation. Significantly, 2.5 μM of ATRi was sufficient to overcome the checkpoint, reversing the phosphorylation of CDK1^Tyr15^ and histone H3^Ser10^. We also tracked the fate of the ATRi-treated cells directly using time-lapse microscopy. Fig 2B shows that while control cells entered and exited mitosis randomly during the imaging period, the majority of cells stopped cell cycle progression and remained in interphase after IR was applied. Significantly, the IR-treated cells were able to enter mitosis in the presence of ATRi, indicating that the G_2_ DNA damage checkpoint was abrogated. As expected, checkpoint abrogation resulted in mitosis that was longer than normal and with frequent mitotic slippage. As a control and in accordance with the above data, incubating the cells with the same concentration of ATRi alone did not affect the unperturbed mitosis (the slight extension of mitosis compare to control was not significant; *P* > 0.1).

Taken together, these results revealed fundamental differences among the current generations of chemicals that target components of the ATR–CHK1–WEE1 kinase cascade: while mitotic catastrophe is induced by targeting either CHK1 or WEE1, unstressed cells are relatively unresponsive to ATR inhibition.

### Partial inhibition of WEE1 and CHK1 has the unexpected effect of promoting cell proliferation

**Figure 3:**
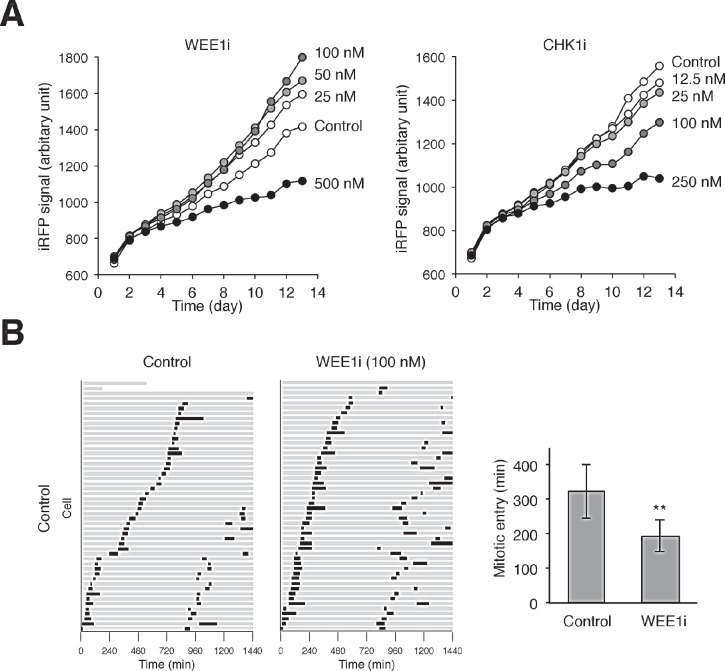
Partial inhibition of WEE1 accelerates the cell cycle (A) Low concentrations of WEE1i stimulates cell growth. HONE1 expressing iRFP (~200 cells) were seeded onto 6-well culture plates and cultured in the presence of buffer or the indicated concentrations of WEE1i or CHK1i (diluted with buffer). After 24 h, the cells were washed gently and propagated in normal medium. The plate was scanned daily with an Odyssey infrared imaging system and the iRFP signal was quantified. Dilution of DMSO alone did not affect cell growth (data not shown). (B) Acceleration of the cell cycle by a low concentration of WEE1i. HONE1 cells expressing histone H2B-GFP were incubated with either buffer or WEE1i (100 nM). Individual cells were then tracked for 24 h with time-lapse microscopy. Each horizontal bar represents one cell (*n* = 50). Grey: interphase; black: mitosis (from DNA condensation to anaphase); truncated bars: cell death. The second mitosis represents that of one of the daughter cells from the first mitosis. The time of entry into the first mitosis was quantified (mean ±90% CI; *n* = 50). WEE1i significantly shortened the time for entering mitosis (** *P* < 0.01; Student's *t-*test).

To determine the effects of different concentrations of WEE1i/CHK1i on cell proliferation, we initially analyzed a cell line (HONE1) expressing the infrared fluorescent protein iRFP. We used the recently devised iRFP-based platform to measure cell proliferation because of its broad linear range, high sensitivity, and the capability in providing rapid and economical time-dependent measurement of the effects of drugs on cell growth [22]. As anticipated, high concentrations of WEE1i and CHK1i inhibited cell proliferation (Fig 3A). We noted, however, that cell growth was slightly increased at lower concentrations of WEE1i. This growth simulation was also observed when the cell cycle of individual cells was analyzed using time-lapse microscopy (Fig 3B). When WEE1i was applied to a randomly growing population, the first mitosis in general occurred earlier than in the control cells. More WEE1i-treated cells were also able to undergo a second mitosis during the imaging period.

A similar increase in cell proliferation after incubation with low concentrations of WEE1i was also observed with other cell lines, including H1299 (Fig S3A) and HeLa (Fig S3B). In some cell lines, an increase in cell growth was also observed with low concentrations of CHK1i (Fig S3A and S3B). We also verified the increase of cell number using conventional trypan blue staining and cell counting (Fig S3C).

Although ATRi alone did not trigger mitotic catastrophe in HeLa cells, it also accelerated the cell cycle (Fig 2B). ATRi-treated cells entered the first mitosis significantly faster (*P* < 0.01; Student's *t-*test) and more cells were able to undergo the second mitosis during the imaging period.

**Figure 4:**
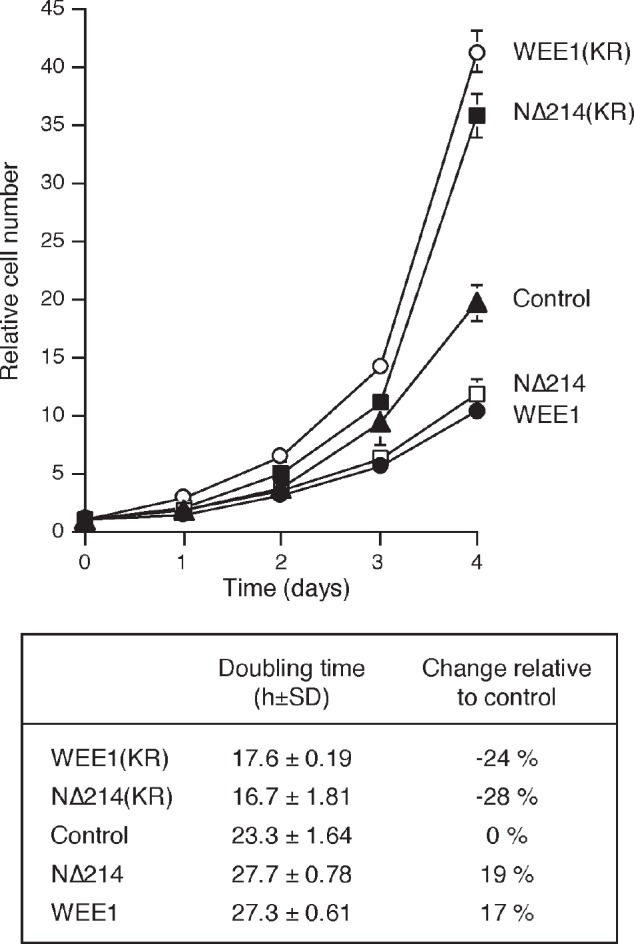
Kinase-inactive WEE1 accelerates the cell cycle The parental H1299 and H1299 expressing WEE1, NΔ214, WEE1(KR), and NΔ214(KR) were plated at a low density. The number of cells were counted at different time points (average ±SD of three independent experiments). The population doubling time was estimated by plotting the log of cell number against time (lower panel).

To further test the idea that partial inhibition of WEE1 could increase cell growth, we generated cell lines stably expressing a kinase-inactive version of WEE1 (K328R) (Fig S4B). A NΔ214 truncation version lacking the negative regulatory domain of WEE1 was also used (see Fig S4A for a schematic diagram of the constructs). Fig 4 shows that both kinase-inactive WEE1^(KR)^ and WEE1^NΔ214(KR)^ speeded up proliferation, reducing the doubling time by over 20%. Although the proportion of different cell cycle phases was not significantly affected by kinase-dead WEE1^(KR)^ (Fig S4C), the cell cycle as a whole was shortened (Fig S4D). By contrast, expression of the corresponding kinase-active WEE1 and WEE1^NΔ214^ reduced cell proliferation (Fig 4) and slowed down the cell cycle (Fig S4D). Taken together, these results indicated that while complete inhibition of WEE1 inhibited cell growth, limited inhibition of the ATR-CHK1-WEE1 pathway, either with relative low concentrations of inhibitors or kinase-inactive mutants of WEE1, could accelerate the cell cycle and stimulate growth.

### Targeting CHK1 and WEE1 together enhances mitotic catastrophe

A general approach for target therapies is to utilize minimum drug doses to reduce non-specific effects and general toxicity. However, as depicted above, limited inhibition of WEE1/CHK1 has the potential of actually stimulating cell proliferation. As ATR, CHK1, and WEE1 are components of a linear pathway, we next tested if more extensive mitotic catastrophe can be induced when more than one component are targeted together, even with relatively low concentrations of the drugs.

**Figure 5:**
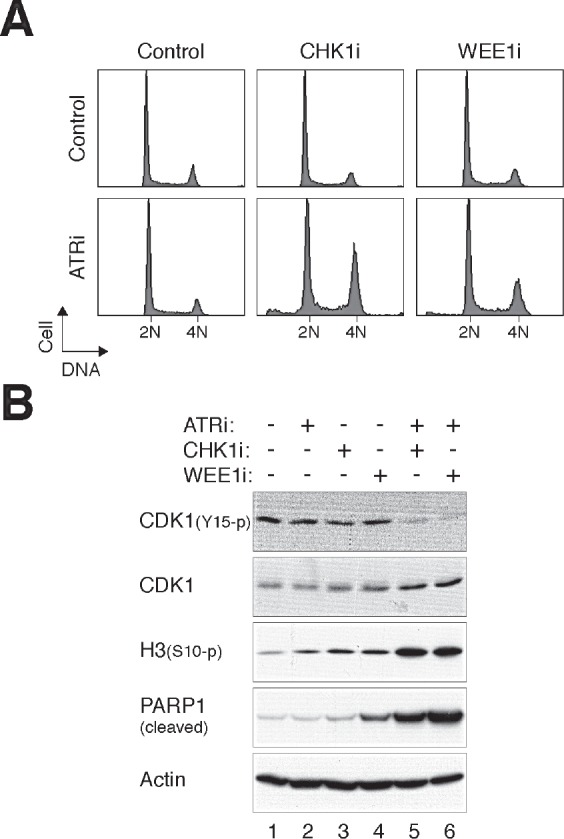
ATRi cooperates with CHK1i/WEE1i in promoting mitotic catastrophe (A) Targeting ATR and CHK1/WEE1 together increases the G_2_/M population. HeLa cells were incubated with different combinations of ATRi (2.5 μM), CHK1i (62.5 nM), and WEE1i (125 nM). After 24 h, the cells were harvested and analyzed with flow cytometry. (B) Targeting ATR and CHK1/WEE1 together promotes mitotic catastrophe. Cells were treated as described in panel (A). Lysates were prepared and the indicated proteins were detected with immunoblotting.

Interestingly, although ATRi alone did not affect the cell cycle (Fig 1A, S1A, and 2B), it was able to enhance premature mitosis when combined with sublethal concentrations of CHK1i or WEE1i. Incubating cells with ATRi and CHK1i or ATRi and WEE1i induced a higher portion of G_2_/M cells than the individual chemicals alone (Fig 5A). Consistent with mitotic catastrophe, cells receiving both chemicals expressed dephosphorylated CDK1^Tyr15^, phosphorylated histone H3^Ser10^, and cleaved PARP1 (Fig 5B).

**Figure 6:**
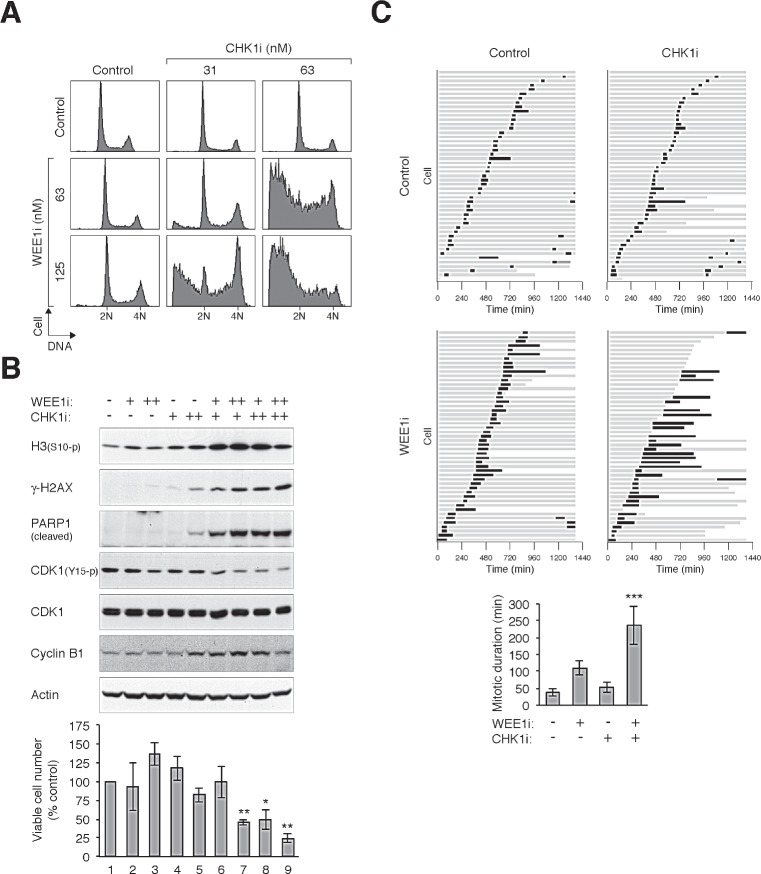
CHK1i cooperates with WEE1i in promoting mitotic catastrophe (A) Combining CHK1i and WEE1i induces extensive cell cycle disruption. HeLa cells were exposed to the indicated concentrations of CHK1i and WEE1i individually or in combination. After 24 h, the cells were harvested and analyzed with flow cytometry. (B) Combining CHK1i and WEE1i induces mitotic catastrophe. Cells were treated as described in panel (A). Lysates were prepared and analyzed with immunoblotting. Uniform loading of lysates was confirmed by immunoblotting for actin. The cells were also harvested for trypan blue exclusion assay (bottom panel, average ±SD of triplicated counting). Combination of CHK1i and WEE1i reduced viability (** *P* < 0.01; * *P* < 0.01; Student's *t-*test). (C) Co-inhibition of CHK1 and WEE1 promotes extensive mitotic delay and cell death. HeLa cells expressing histone H2B-GFP were incubated with CHK1i (100 nM) or WEE1i (100 nM) individually or in combination. Individual cells were then tracked for 24 h with time-lapse microscopy. Each horizontal bar represents one cell (*n* = 50). Grey: interphase; black: mitosis (from DNA condensation to anaphase); truncated bars: cell death. The mitotic duration was quantified (mean ±90% CI) (*** *P* < 0.001; Student's *t-*test).

By comparison, combining CHK1i and WEE1i promoted massive cell death (Fig 6A). Again, sublethal concentrations of CHK1i and WEE1i which did not trigger mitotic catastrophe on their own were used here. The combined treatments prompted dephosphorylation of CDK1^Tyr15^, phosphorylation of histone H3^Ser10^, and accumulation of γ-H2AX (Fig 6B). Consistent with apoptosis, there was an increase in PAPR1 cleavage and cell death (Fig 6B). Time-lapse microscopy confirmed that CHK1i and WEE1i together induced a prolonged mitosis and extensive cell death compare to the individual chemicals alone (Fig 6C).

**Figure 7:**
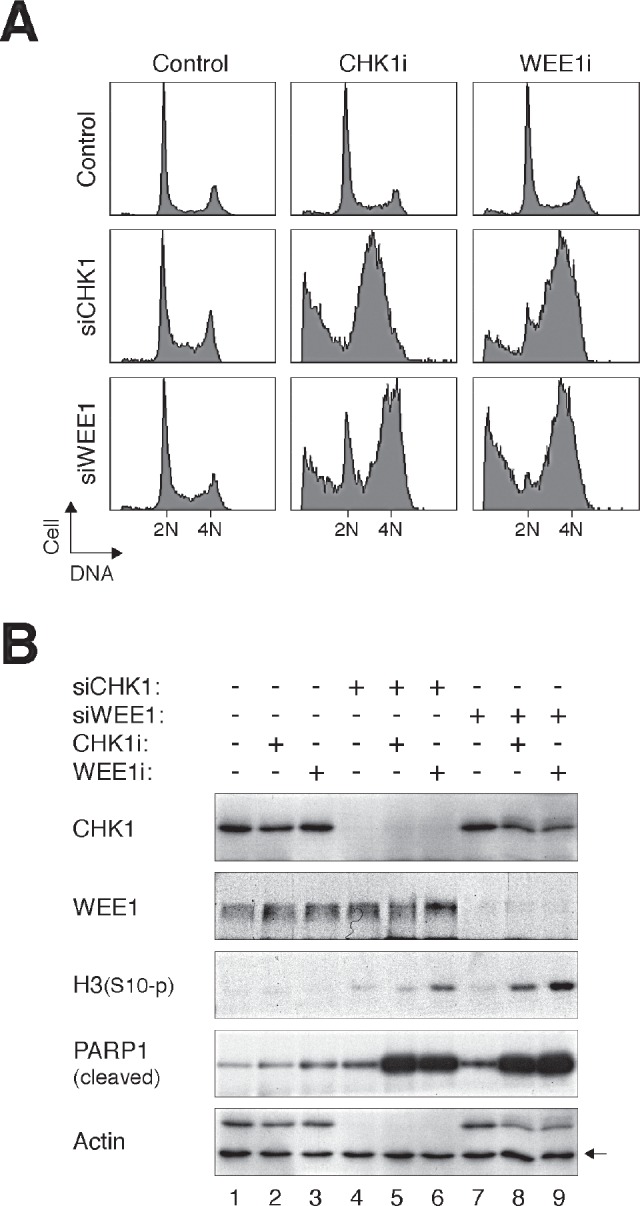
Depletion of CHK1 or WEE1 increases the sensitivity to CHK1i and WEE1i (A) HeLa cells were transfected with either control, siCHK1, or siWEE1 (1.25 nM). After 24 h, the cells were incubated with either CHK1i (31.25 nM) or WEE1i (62.5 nM) for another 24 h. The cells were then harvested and analyzed with flow cytometry. (B) HeLa cells were treated as in panel (A). Lysates were prepared and the indicated proteins were analyzed with immunoblotting. Uniform loading of lysates was confirmed by immunoblotting for actin (the upper band in the actin panel is CHK1).

To address if the effects of CHK1i and WEE1i were specific for the respective kinases, CHK1 and WEE1 were also downregulated with siRNAs. Fig 7A verifies that downregulation of CHK1 with siRNA increased the sensitivity to WEE1i (Fig 7A). Conversely, siRNA against WEE1 enhanced the disruption of the cell cycle by CHK1i. Not surprisingly, knockdown of CHK1 or WEE1 enhanced the effectiveness of CHK1i or WEE1i, respectively. Immunoblotting analysis confirmed the downregulation of CHK1 and WEE1 (Fig 7B). It also revealed the increase in premature mitosis (histone H3^Ser10^ phosphorylation) and apoptosis (PARP1 cleavage) by the combination of siRNAs and inhibitors.

Collectively, these data indicated that even with sublethal concentrations of inhibitors, targeting ATR with either CHK1 or WEE1, or CHK1 and WEE1 concurrently induced massive mitotic catastrophe.

## DISCUSSION

A major focus of the clinical development of inhibitors of the ATR–CHK1–WEE1 pathway is for combination with chemo- and radiotherapy. For example, ATRi (VE-821) was found to enhance the cytotoxicity caused by DNA damaging agents, particularly in cells with defective ATM and p53 [23]. Likewise, several studies have detailed the properties of inhibitors of CHK1 [24] and WEE1 [25] on sensitizing cells to DNA damage.

As standalone agents, CHK1i and WEE1i are believed to induce DNA damage by unscheduled initiation of DNA replication [16][18]. Given that CHK1 and WEE1 are components of the checkpoint itself, the DNA damage induced by CHK1i/WEE1i is unable to elicit an effective checkpoint response. Hence inhibition of CHK1/WEE1 is expected to disrupt cells in a two-step process. DNA damage is first induced by the unscheduled initiation of DNA replication during S phase, which normally would turn on the G_2_ DNA damage checkpoint. The presence of CHK1i/WEE1i, however, uncoupled the checkpoint and allowed the damaged cells to enter mitosis. It should be noted that the cell lines used in this study have defective p53 responses (HeLa: p53 is degraded by HPV E6; H1299: p53 genes are deleted), a feature commonly found in many cancers. The lack of p53-dependent cell cycle arrest should further enhance both the precocious S phase progression and mitotic entry induced by CHK1i and WEE1i.

In agreement with the above hypothesis, CHK1i and WEE1i induced an accumulation of G_2_/M cells in HeLa (Fig 1A) and H1299 (Fig S1A). Several lines of evidence verified that CHK1i and WEE1i triggered mitotic catastrophe, including the increase of histone H3^Ser10^ phosphorylation, apoptosis (Fig 1B and 1C), and an extension of mitotic duration (Fig 1D). Not surprisingly, exposure to CHK1i/WEE1i eventually led to a decline of viability (Fig 3A and S3), probably mainly due to mitotic cell death (Fig 1D and 1E).

By comparing inhibitors targeting ATR, CHK1, and WEE1 side-by-side on the same cell lines, we found that in contrast to CHK1i and WEE1i, ATRi was relatively ineffective in inducing mitotic catastrophe (Fig 1A). These findings were confirmed more rigorously by tracking individual cells using live-cell imaging (Fig 2B and S2). Moreover, the relative ineffectiveness of ATRi was not limited to HeLa cells (Fig 1A), but also displayed by H1299 (Fig S1A), as well as several cell lines from nasopharyngeal carcinoma we tested (our unpublished data). Given that the K_i_ of the ATRi VE-821 is 6 nM (and >600-fold selectivity over related kinases ATM or DNA-PK) [21], the concentrations of ATRi used in this study should be sufficient to inhibit the kinase. Indeed, the IR-induced G_2_ DNA damage checkpoint could readily be uncoupled with ATRi, leading to dephosphorylation of CDK1^Tyr15^ and precocious mitotic entry (Fig 2). Although the mechanistic basis of the relatively weak cytotoxicity of ATRi compare to CHK1i/WEE1i remains to be defined, our observations suggest that targeting different components of the ATR–CHK1–WEE1 pathway may not be equally effective in the same cell system.

We observed that instead of reducing proliferation, low concentrations of CHK1i and WEE1i actually increased cell number (Fig 3 and S3). These data indicated that the disruption of cell cycle control after partial inhibition of CHK1/WEE1 did not necessary result in lethal mitotic catastrophe. Although the increase in cell growth occurred in a narrow concentration range of CHK1i/WEE1i and is probably cell-type dependent, this has broad clinical implications on the use of small-molecule inhibitors of the ATR–CHK1–WEE1 pathway. This is likely to be caused by the shortening of the cell cycle in the cancer cells after treatment with low concentrations of the inhibitors (Fig 4). As cancer cells including H1299 and HeLa are already highly aneuploid, they are likely to be relatively tolerant to some degree of DNA damage or chromosomal instability associated with the acceleration of cell cycle caused by partial inhibition of WEE1 pathway.

Given the potential detrimental effects of partial inhibition of the ATR–CHK1–WEE1 pathway, it is important to understand how the balance can be offset towards mitotic catastrophe. We demonstrated that one possible solution of this problem is by targeting more than one components of the pathway together. Although ATRi was not effective on its own, it could enhance the mitotic catastrophe induced by sublethal concentrations of CHK1i and WEE1i (Fig 5). Furthermore, challenging cells with CHK1i and WEE1i together induced more extensive mitotic catastrophe than the individual drugs alone (Fig 6). These results are consistent with the synergistic effects of inhibitors of CHK1 and WEE1 observed in other cancer models. For example, WEE1i (MK-1775) cooperates with the CHK1 inhibitor AR458323 in inhibiting cell proliferation in prostate and lung cancer cell lines [26]. Another CHK1 inhibitor PF-00477736 acts synergistically with WEE1i in a panel of cancer cell lines (including breast, colon, ovarian, and prostate) [27]. The CHK1 inhibitor MK-8776 also cooperates with WEE1i in reducing tumor growth in colorectal, ovarian [28], and neuroblastoma [29] mouse xenograft models. Together, these data indicated that although CHK1i/WEE1i have the potential drawback of enhancing cancer cell growth at low concentrations, targeting more than one component of the checkpoint pathway together can help to tip the balance towards mitotic catastrophe.

## MATERIALS AND METHODS

### DNA constructs

Plasmid expressing iRFP [30] was obtained from Addgene (Cambridge, MA, USA). Plasmid expressing iFP1.4 [31] was a gift from Roger Tsien (University of California, San Diego). Histone H2B-GFP construct was a gift from Tim Hunt (Cancer Research UK). WEE1, WEE1^(K328R)^, WEE1^NΔ214^, and WEE1^NΔ214+(K328R)^ in pSLX-CMV were generous gifts from Nobumoto Watanabe (RIKEN, Japan). WEE1 cDNA was amplified using primers 5′-CGCCATGGGCTTCCTGAGCCGACAGCAGC-3′ and 5′-TCACTCGAGGTATATAGTAAGGCTGA-3′. The PCR product was cut with *Nco* I-*Xho* I and ligated into pGEX-KG to create GST-WEE1 in pGEX-KG. The *Nco* I-*Hind* III fragment from GST-WEE1 in pGEX-KG was put into pUHD-P3 [32] to generate FLAG-WEE1 in pUHD-P3.

### Cell culture

H1299 (non-small cell lung carcinoma) and HeLa (cervical carcinoma) were obtained from the American Type Culture Collection (Manassas, VA, USA). The HeLa used in this study was a clone that expressed the tTA tetracycline repressor chimera [33]. The nasopharyngeal carcinoma cell line HONE1 [34] was obtained from NPC AoE Cell Line Repository (The University of Hong Kong). Cells were propagated in Dulbecco's modified Eagle's medium (DMEM) supplemented with 10% (v/v) calf serum (Life Technologies, Carlsbad, CA, USA) (for HeLa) or 10% (v/v) fetal bovine serum (for other cell lines) and 50 U/ml penicillin-streptomycin (Life Technologies).

HeLa cells stably expressing histone H2B-GFP [35] were used for live-cell imaging. H1299, HeLa, and HONE1 cells expressing iRFP were generated by transfection followed by cell sorting. The cells were transfected with an iRFP-expressing construct and iRFP-positive cells were enriched by sorting using a flow cytometer with a 633-nm red laser for excitation (FACSAria II, Becton Dickinson, Franklin Lakes, NJ, USA). The cells were sorted again after one week. Three rounds of sorting were performed.

Cell lines expressing recombinant WEE1 were produced by transfecting constructs of pSLX-CMV expressing WEE1, WEE1^NΔ214^, WEE1^(K328R)^, or WEE1^NΔ214(K328R)^ into H1299 cells. The cells were then selected in medium supplemented with 100 μg/ml of G418. Medium containing G418 was replenished every three days and individual colonies were isolated and expanded in culture after about 3 weeks of selection. Cell-free extracts were prepared and the expression of WEE1 or mutants was analyzed by immunoblotting. After the establishment of the cell lines, subsequent experiments were performed in the absence of G418. Cell growth of WEE1-expressing cells was measured by plating the cells at a density of about 10,000 cells/60-mm plate, and counting the attached cells in the same randomly selected areas (five 2-mm diameter circles) every 24 h using a light microscope. The positions of the circles were fixed at the bottom of the culture plate.

Unless stated otherwise, cells were treated with the following reagents at the indicated final concentration: AZD7762 (Selleck Chemicals), MK-1775 (Selleck Chemicals), nocodazole (Sigma-Aldrich, St. Louis, MO, USA; 0.1 μg/ml), thymidine (Sigma-Aldrich; 2 mM), and VE-821 (Selleck Chemicals; 2.5 μM). Double thymidine synchronization [36], trypan blue analysis [37] and preparation of cell-free extracts [38] were performed as previously described.

### RNA interference

Unless stated otherwise, cells were transfected with siRNA (1.25 nM) using Lipofectamine^TM^ RNAiMAX (Life Technologies). Stealth siRNA targeting CHK1 (GGCUUGGCAACAGUAUUUCGGUAUA) and WEE1 (CCUCAGGACAGUGUCGUCGUAGAAA) were obtained from Life Technologies.

### Flow cytometry

Flow cytometry analysis after propidium iodide staining was performed as described previously [37].

### Infrared imaging

Infrared images of cells expressing iRFP were acquired and quantified with an Odyssey CLx system (LI-COR Biosciences, Lincoln, NE, USA).

### Live-cell imaging

Cells were seeded onto 24-well culture plates and imaged using a Ti-E inverted fluorescent microscope (Nikon, Tokyo, Japan) equipped with a SPOT BOOST EMCCD camera (Diagnostic Instrument, Sterling Heights, MI, USA) and a INU-NI-F1 temperature, humidity, and CO_2_ control system (Tokai Hit, Shizuoka, Japan). Data acquisition was carried out at 5 min/frame.

### Antibodies and immunological methods

Antibodies against CDK1 [39] and cyclin B1 [35] were obtained from sources as described previously. Antibodies against β-actin (Sigma-Aldrich), γH2AX (Bethyl Laboratories, Montgomery, TX, USA), CHK1, phospho-histone H3^Ser10^, and WEE1 (Santa Cruz Biotechnology, Santa Cruz, CA, USA), phospho-CDK1^Tyr15^ and cleaved PARP1(Asp214) (BD Biosciences, Franklin Lakes, NJ, USA) were obtained from the indicated suppliers. Immunoblotting was performed as described previously [38].

### Statistical Analysis

Statistical analyses were performed, and graphs were generated using Excel (Microsoft).

## SUPPLEMENTARY INFORMATION FIGURES


