# Supplementary material for: Pharmacological targeting the ATR–CHK1–WEE1 axis involves balancing cell growth stimulation and apoptosis
Source: Oncotarget. 2014 Sep 25;5(21):10546–57. doi: 10.18632/oncotarget.2508 (PMC4279392; doi:10.18632/oncotarget.2508)
Supplement: Supplementary file 1 [file oncotarget-05-10546-s001.pdf]

Pharmacological targeting the ATR–CHK1–WEE1 axis involves balancing cell growth stimulation and apoptosis

Supplementary Information

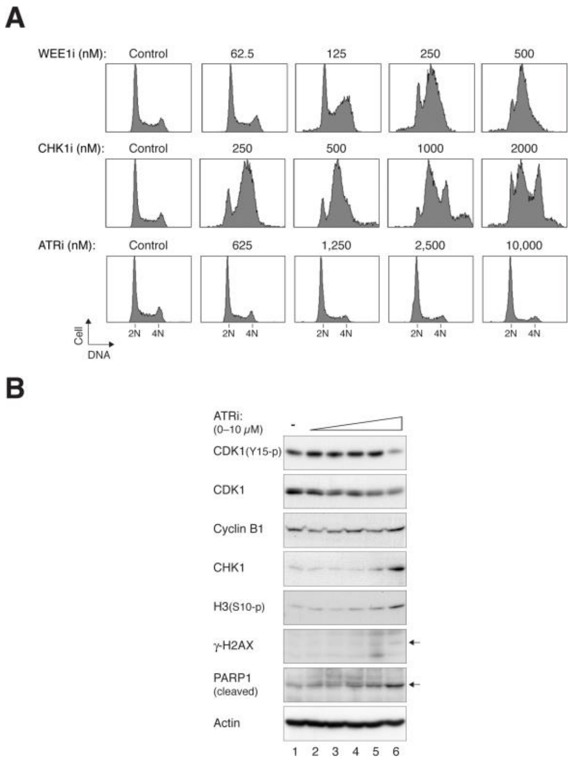

Figure S1: Treatment with ATRi alone does not affect the cell cycle. (A) Inhibition of CHK1, WEE1, but not ATR disrupts the cell cycle in H1299. H1299 cells were incubated with either buffer or the indicated concentrations of MK-1775 (WEE1i), AZD7762 (CHK1i), or VE-821 (ATRi). After 24 h, the cells were harvested and analyzed with flow cytometry. The positions of 2N and 4N DNA content are indicated. **(B)** ATRi does not induce mitotic catastrophe. HeLa cells were incubated with either buffer or increasing concentrations of VE-821 (ATRi) (lanes 2–6: 625 nM, 1.25 µM, 2.5 µM, 5 µM, 10 µM) for 24 h. Lysates were prepared and the expression of the indicated proteins was analyzed with immunoblotting. Actin analysis was included to assess protein loading and transfer.

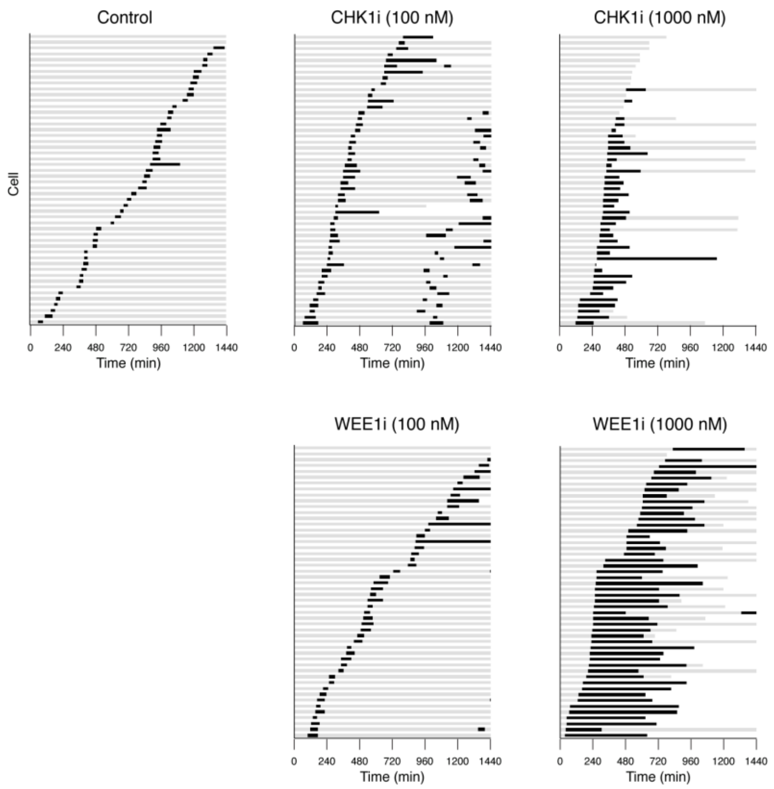

Figure S2: Inhibition of CHK1 and WEE1 increases mitotic duration and triggers cell death. HeLa cells expressing histone H2B-GFP were incubated with CHK1i or WEE1i (100 nM or 1 µM). Individual cells were then tracked for 24 h using time-lapse microscopy. Each horizontal bar represents one cell (n = 50). Grey: interphase; black: mitosis (from DNA condensation to anaphase or mitotic slippage); truncated bars: cell death.

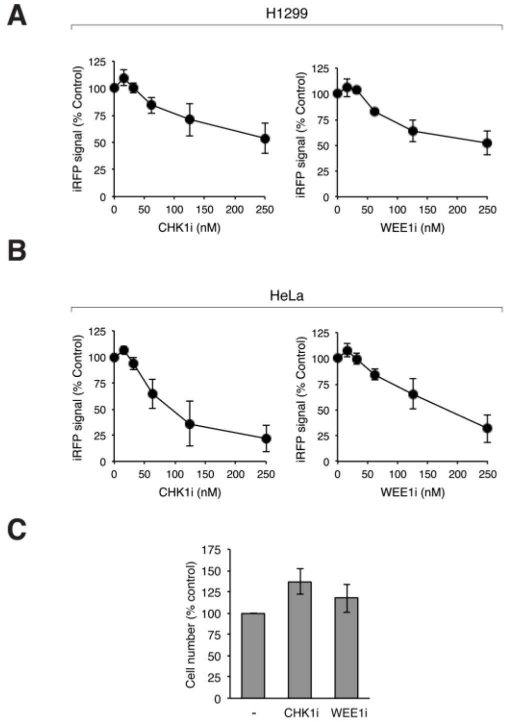

Figure S3. Partial inhibition of WEE1 increases cell proliferation. (A) The effects of CHK1i and WEE1i on cell proliferation in H1299. H1299 cells expressing iRFP (~200 cells) were seeded onto 6-well culture plates and cultured in the presence of buffer or different concentrations of WEE1i or CHK1i. After 72 h, the plate was scanned with an Odyssey infrared imaging system and the iRFP signal was quantified (mean ±SD of triplicates). **(B)** The effects of CHK1i and WEE1i on cell proliferation in HeLa. HeLa cells expressing iRFP (~200 cells) were seeded onto 6-well culture plates and grown in the presence of buffer or different concentrations of WEE1i or CHK1i. After 72 h, the plate was scanned with an Odyssey infrared imaging system and the iRFP signal was quantified (mean ±SD of triplicates). **(C)** Low concentrations of CHK1i and WEE1i increase cell proliferation in HeLa. HeLa cells were incubated with control buffer, CHK1i (62.5 nM), or WEE1i (62.5 nM). After 24 h, the number of viable cells were analyzed using trypan blue staining and a haemocytometer (mean ±SD of three independent experiments).

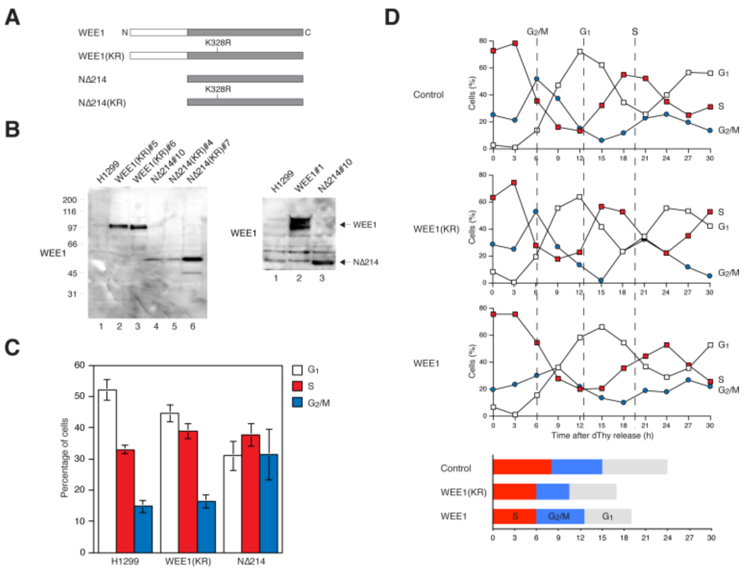

Figure S4. Expression of wild type and kinase-dead mutants of WEE1. (A) Schematic diagram of WEE1 and mutant proteins used in this study. The C-terminal catalytic domain of WEE1 is highlighted in grey. The position of the K328R mutation, which results in catalytic-inactive WEE1 is indicated. **(B)** Generation of WEE1-expressing cells. Cells lines stably expressing wild type and mutant WEE1 were created as described in Materials and Methods. Lysates from the parental H1299 (lane 1) and H1299 expressing WEE1, WEE1(KR), NΔ214, or NΔ214(KR) were prepared. The expression of the recombinant WEE1 was analyzed by immunoblotting using antibodies against WEE1. Molecular size standards (in kDa) are indicated on the left. **(C)** Cell cycle distribution of cells overexpressing wild type and kinase-inactive WEE1. Asynchronously growing parental H1299 and H1299 expressing WEE1(KR) or NΔ214 were fixed and analyzed with flow cytometry. The percentage of cells in different phases of the cell cycle is shown (average ±SD of three independent experiments). **(D)** Kinase-inactive WEE1 accelerates cell cycle progression. The parental H1299 or H1299 expressing WEE1 or WEE1(KR) were blocked in early S phase with a double thymidine method and released into the cell cycle. Cells

harvested at different time points were analyzed with flow cytometry. The approximate time of the G_2/M, G_1, and S phase of the parental H1299 cells are indicated by the dotted lines and plotted at the bottom.
